# Magnetoliposomes containing magnesium ferrite nanoparticles as nanocarriers for the model drug curcumin

**DOI:** 10.1098/rsos.181017

**Published:** 2018-10-17

**Authors:** Beatriz D. Cardoso, Irina S. R. Rio, Ana Rita O. Rodrigues, Francisca C. T. Fernandes, B. G. Almeida, A. Pires, A. M. Pereira, J. P. Araújo, Elisabete M. S. Castanheira, Paulo J. G. Coutinho

**Affiliations:** 1 Centro de Física (CFUM), Universidade do Minho, Campus de Gualtar, 4710-057 Braga, Portugal; 2 IFIMUP/IN - Instituto de Nanociência e Nanotecnologia, R. Campo Alegre, 4169-007 Porto, Portugal

**Keywords:** magnesium ferrite nanoparticles, magnetoliposomes, curcumin, superparamagnetism, pegylation, cancer therapy

## Abstract

Magnesium ferrite nanoparticles, with diameters around 25 nm, were synthesized by coprecipitation method. The magnetic properties indicate a superparamagnetic behaviour, with a maximum magnetization of 16.2 emu g^−1^, a coercive field of 22.1 Oe and a blocking temperature of 183.2 K. These MgFe_2_O_4_ nanoparticles were used to produce aqueous and solid magnetoliposomes, with sizes below 130 nm. The potential drug curcumin was successfully incorporated in these nanosystems, with high encapsulation efficiencies (above 89%). Interaction by fusion between both types of drug-loaded magnetoliposomes (with or without PEGylation) and models of biological membranes was demonstrated, using FRET or fluorescence quenching assays. These results point to future applications of magnetoliposomes containing MgFe_2_O_4_ nanoparticles in cancer therapy, allowing combined magnetic hyperthermia and chemotherapy.

## 1. Introduction

Magnetic nanoparticles with superparamagnetic properties have attracted increased attention for applications in biomedicine, as they exhibit a strong magnetization only when an external magnetic field is applied [1–3]. Magnetoliposomes (liposomes entrapping magnetic nanoparticles) are promising therapeutic systems which can be guided and localized to specific sites by external magnetic field gradients [3–5] and used as an alternative to conventional chemotherapy through magnetically controlled drug delivery and hyperthermia [6–9].

In the past few years, rising attention has been given to the synthesis of ferrite nanoparticles without transition metals. Calcium and magnesium have been proposed to substitute transition metals in the crystalline structure of ferrite nanoparticles, as their ions are safely metabolized by the human body and may promote a higher biocompatibility [9,10]. In fact, *in vitro* cytotoxicity tests on T-cell lines showed that calcium ferrite nanoparticles are biocompatible at concentrations below 250 mg ml^−1^, exhibiting an enhanced cell viability when compared to other ferrites [11].

Magnesium ferrite nanoparticles are less sensitive to oxidation than magnetite and can be used as hyperthermia agents, as they display a large heating capacity which generates appropriate temperatures for hyperthermia [12,13]. Among all the techniques available for the synthesis of magnesium ferrite nanoparticles, the most used are sol-gel, combustion and coprecipitation methods [14–17]. In order to obtain smaller particles, reverse coprecipitation and reverse micelle methods have been proposed [14,18,19], to achieve nanoparticles with crystallite diameters around 20 nm [14,18].

Curcumin is a natural polyphenolic compound with anti-inflammatory, antioxidant, antimicrobial and anti-cancer properties [20]. It was demonstrated that this compound has been involved in the mechanisms of regulation of target molecules, including transcription factors, growth factors, cytokines, enzymes and several genes that regulate cell proliferation and apoptosis [21]. Considering these properties and the lack of toxicity at high doses, curcumin has gained increased attention as a potential therapeutic agent for cancer, diabetes, allergies, neurodegenerative disorders, arthritis and other inflammatory diseases [22–24]. However, the therapeutic use of curcumin has been limited due to its hydrophobic character, implying low solubility and stability in aqueous media, and poor bioavailability (reduced absorption, rapid metabolism and systemic elimination). These obstacles have hampered the verification of the therapeutic efficacy of curcumin in clinical trials [25]. In order to overcome these limitations, the use of drug delivery systems, such as polymeric nanoparticles, micelles, liposomes and hydrogels, has been increased, to improve the bioavailability of the compound [24–30].

In this work, novel magnetoliposomes containing biocompatible magnesium ferrite nanoparticles were tested as nanocarriers for curcumin as a model drug. These studies have demonstrated promising results for the use of these magnetoliposomes as drug nanocarrier systems, with potential applications in cancer therapy.

## 2. Experimental procedure

All the solutions were prepared using spectroscopic grade solvents and ultrapure water (Milli-Q grade).

### 2.1. Preparation of magnesium ferrite nanoparticles

Magnesium ferrite nanoparticles were prepared by coprecipitation method. First, 10 ml of an aqueous solution containing 1.08 g of magnesium sulfate, 1.39 g of iron(II) sulfate heptahydrate and 200 µl of a 10% sulfuric acid solution were heated at 75°C, under magnetic stirring, until a clear solution was obtained. Then, 1.02 g of potassium oxalate monohydrate were dissolved in 15 ml of warm deionized water. The two solutions were then mixed, under vigorous stirring, at 90°C. After 15 min, the solution was cooled to room temperature. The precipitated nanoparticles were washed by several cycles of centrifugation and redispersion in water. Finally, the nanoparticles were calcined at 600°C for 3 h.

### 2.2. Preparation of magnetoliposomes

For magnetoliposomes preparation, the lipids egg yolk phosphatidylcholine (Egg-PC), dipalmitoyl-phosphatidylcholine (DPPC) (from Sigma-Aldrich) and 1,2-distearoyl-*sn*-glycero-3-phosphoethanolamine-*N*-[methoxy(polyethylene glycol)-2000] (ammonium salt) (DSPE-PEG2000, from Avanti Polar Lipids) were used. For aqueous magnetoliposomes (AMLs) preparation, a 10 mM lipid solution in ethanol was injected, under vigorous vortexing, to a dispersion of magnetic nanoparticles in ultrapure water (pH = 7), above the melting transition temperature of the lipids, 41°C for DPPC and −18°C for Egg-PC (ethanolic injection method) [31]. At this pH, the nanoparticles are below their point of zero charge (a PZC value of 8.4 was reported for similar MgFe_2_O_4_ nanoparticles also calcined at 600°C) [32] and thus are expected to be well dispersed at the time of injection. After encapsulation, an ultracentrifugation was performed to remove all the non-encapsulated nanoparticles (NPs).

Solid magnetoliposomes (SMLs) were prepared by a method previously developed [33,34]. First, 10 µl of the synthesized MgFe_2_O_4_ NPs were dispersed in 3 ml of water and centrifuged. Then, the deposited particles were dispersed in 10 µl water in an ultrasonicator, for 1 min at 189 W, and 3 ml of chloroform were added to the aqueous dispersion of NPs. After vigorous agitation, 165 µl of a 20 mM solution of dipalmitoylphosphatidylcholine (DPPC) were added under vortexing, to form the first lipid layer of the SMLs. The particles were washed twice by magnetic decantation with ultrapure water, in order to remove the lipid that was not attached to the NPs. The second lipid layer was then formed by the injection of 165 µl of a lipid solution (20 mM), under vortexing, in a 3 ml aqueous dispersion of the particles with the first layer. The resulting SMLs were then washed and purified with ultrapure water by centrifugation. The formation of a lipid bilayer around the prepared magnesium ferrite nanoparticles was confirmed by Förster resonance energy transfer (FRET) measurements, using the labelled lipids NBD-C_12_-HPC (1-palmitoyl-2-{12-[(7-nitro-2-1,3-benzoxadiazol-4-yl)amino]hexanoyl}-*sn*-glycero-3-phosphocholine) and Rhodamine B-DOPE (*N*-(lissamine Rhodamine B sulfonyl)-1,2-dioleoyl-*sn*-3-phosphatidylethanolamine (ammonium salt)), both from Avanti Polar Lipids (structures in electronic supplementary material, figure S1), as previously described [33,34].

Curcumin was entrapped in AMLs by the co-injection method, while in solid magnetoliposomes the drug was incorporated by injection of an ethanolic solution together with the formation of the second lipid layer, as already reported for the incorporation in magnetoliposomes of several new anti-tumour compounds [34–36].

### 2.3. Preparation of giant unilamellar vesicles

Soya bean lecithin (*L*-α-Phosphatidylcholine), from Sigma-Aldrich, was used for giant unilamellar vesicles (GUVs) preparation, using a procedure described by Tamba *et al*. [37,38]. First, 100 µl of soya bean lecithin solution (1 mM) were dried under an argon stream to produce a thin and homogeneous lipid film. Next, 40 µl of water were added to the film and it was incubated at 45°C for 30 min. Then, 3 ml of 0.1 M glucose aqueous solution were added and the resulting mixture was again incubated at 37°C for 2 h. Finally, after incubation, the GUVs suspension was centrifuged at 14 000*g* for 30 min at 20°C, to remove multilamellar vesicles and lipid aggregates.

### 2.4. Spectroscopic measurements

#### 2.4.1. General methods

Absorption spectra were recorded in a Shimadzu UV-3101PC UV–Vis–NIR spectrophotometer. Fluorescence measurements were performed using a Fluorolog 3 spectrofluorimeter, equipped with double monochromators in both excitation and emission, Glan-Thompson polarizers and a temperature-controlled cuvette holder. Fluorescence spectra were corrected for the instrumental response of the system.

The steady-state fluorescence anisotropy, *r*, taken as the average value in an appropriate spectral range, is calculated by

2.1 $$r=\frac{I_{\mathrm{VV}}-GI_{\mathrm{VH}}}{I_{\mathrm{VV}}+2GI_{\mathrm{VH}}},$$

where *I*_VV_ and *I*_VH_ are the intensities of the emission spectra obtained with vertical and horizontal polarization, respectively (for vertically polarized excitation light), and $G=I_{\mathrm{HV}}/I_{\mathrm{HH}}$ is the instrument correction factor, where *I*_HV_ and *I*_HH_ are the emission intensities obtained with vertical and horizontal polarization (for horizontally polarized excitation light).

#### 2.4.2. FRET measurements

FRET efficiency, *Φ*_RET_, defined as the proportion of donor molecules that have transferred their excess energy to acceptor molecules, can be obtained by taking the ratio of the donor-integrated fluorescence intensities in the presence of acceptor (*F*_DA_) and in the absence of acceptor (*F*_D_) (equation (2.2)) [39]

2.2 $$\Phi_{\;\mathrm{RET}}\;=\;1-\frac{F_{\mathrm{DA}}}{F_{D}}.$$

The distance between donor and acceptor molecules can be determined through the FRET efficiency (equation (2.3))

2.3 $$r_{\mathrm{AD}}=\;R_{0}{\left[\frac{1-\Phi_{\mathrm{RET}}}{\Phi_{\mathrm{RET}}}\right]}^{1/6},$$

where *R*_0_ is the Förster radius (critical distance), that can be obtained by the spectral overlap, *J*(*λ*), between the donor emission and the acceptor absorption, according to equations (2.4) and (2.5) (with *R*_0_ in Å, *λ* in nm, *ε*_A_(*λ*) in M^−1^ cm^−1^), [39]

2.4 $$R_{0\;}=0.2108{\left[k_{D}^{2}{\Phi \;}_{D}^{0}n^{-4}J(\lambda )\right]}^{1/6},$$

and

2.5 $$J(\lambda )=\;\int_{0}^{\infty }I_{D}(\lambda )\varepsilon_{A}(\lambda )\lambda^{4}\;d\lambda ,$$

where $k^{2}=2/3$ is the orientational factor assuming random orientation of the dyes, ${\Phi \;}_{D}^{0}$ is the fluorescence quantum yield of the donor in the absence of energy transfer, *n* is the refraction index of the medium, *I*_D_(*λ*) is the fluorescence spectrum of the donor normalized so that $\int_{0}^{\infty }I_{D}(\lambda )\;d\lambda =1,$ and *ɛ*_A_(*λ*) is the molar absorption coefficient of the acceptor.

For determination of fluorescence quantum yield of NBD-C_12_-HPC (energy donor) in magnetoliposomes containing magnesium ferrite nanoparticles, this fluorescent labelled lipid incorporated in lipid membranes was used as reference, *Φ*_r_ = 0.32 at 25°C, as reported by Invitrogen [40].

### 2.5. Structural characterization

#### 2.5.1. Scanning electron microscopy

SEM images of magnesium ferrite nanoparticles and solid magnetoliposomes were recorded using a scanning electron microscope, FEI Nova 200 NanoSEM, operating in transmission mode (STEM). In the case of SMLs, a negative staining was employed. For that, a 2% aqueous solution of ammonium molybdate tetrahydrate was prepared. Then, 20 µl of sample and 20 µl of staining solution were mixed and a drop of this mixture was placed onto a Formvar grid, held by tweezers. After 20 s, almost all the solution was removed with filter paper and left dry. The processing of STEM images was performed using ImageJ software. It consisted in enhancing local contrast followed by automatic local thresholding and particle analysis. The area of each particle allowed an estimation of the particle diameter. The resulting histograms were fitted to Gaussian distributions.

#### 2.5.2. X-ray diffraction and dynamic light scattering measurements

X-ray diffraction (XRD) analyses were performed using a conventional Philips PW 1710 diffractometer, operating with CuK*_α_* radiation, in a Bragg–Brentano configuration.

Liposomes mean diameter and size distribution (polydispersity index) were measured using dynamic light scattering (DLS) equipment (NANO ZS Malvern Zetasizer) at 25°C, using a He-Ne laser of *λ* = 632.8 nm and a detector angle of 173°. Five independent measurements were performed for each sample.

### 2.6. Magnetic measurements

#### 2.6.1. General methods

Magnetic measurements were performed at room temperature in a Superconducting Quantum Interference Device (SQUID) magnetometer (Quantum Design MPMS5XL), using applied magnetic fields up to 5.5 T.

#### 2.6.2. Temperature dependence of the magnetization and magnetic hysteresis cycles

The temperature dependence of the magnetization was measured in the temperature range from 5 to 380 K. The curves were obtained by initially cooling the sample under an applied magnetic field of *H* = 100 Oe (field cooled, FC) and then measuring its magnetization with increasing temperature (applied field of *H* = 50 Oe). Subsequently, after reaching 350 K, the sample was recooled, this time with no applied magnetic field (zero-field-cooled, ZFC) and the magnetization measurements were again performed with increasing temperature, under the same magnetic field of *H* = 50 Oe. From the behaviour of the FC and ZFC curves, the blocking temperature (*T*_B_) of the superparamagnetic nanoparticles can be obtained [41]. The magnetization hysteresis loop measurements were made by fixing the temperature and measuring the magnetization at a series of different applied magnetic fields. This type of study gives information about the maximum magnetization and the degree at which the sample remains magnetized when the applied field is removed, and how easily the sample magnetization can be reversed, the so-called coercive field.

### 2.7. Curcumin encapsulation efficiency

The encapsulation efficiency, *EE*(%), of curcumin in magnetoliposomes was determined through fluorescence emission measurements. After preparation, drug-loaded magnetoliposomes (MLs) were subjected to centrifugation at 10 000 r.p.m. for 60 min. The supernatant was pipetted out and its fluorescence was measured, allowing to determine the drug concentration using a calibration curve previously obtained. Three independent measurements were performed for each system and standard deviations (s.d.) were calculated. The *EE*(%) was determined using the following equation:

2.6 $$EE(\text{\%})=\frac{\left(\mathrm{total}\;\mathrm{amount}-\mathrm{amount}\;\mathrm{of}\;\mathrm{non}\text{-}\mathrm{encapsulated}\;\mathrm{drug}\right)}{\mathrm{total}\;\;\mathrm{amount}}\times 100\;.$$

## 3. Results and discussion

### 3.1. Nanoparticles characterization

#### 3.1.1. X-ray diffraction analysis

**Figure 1.**
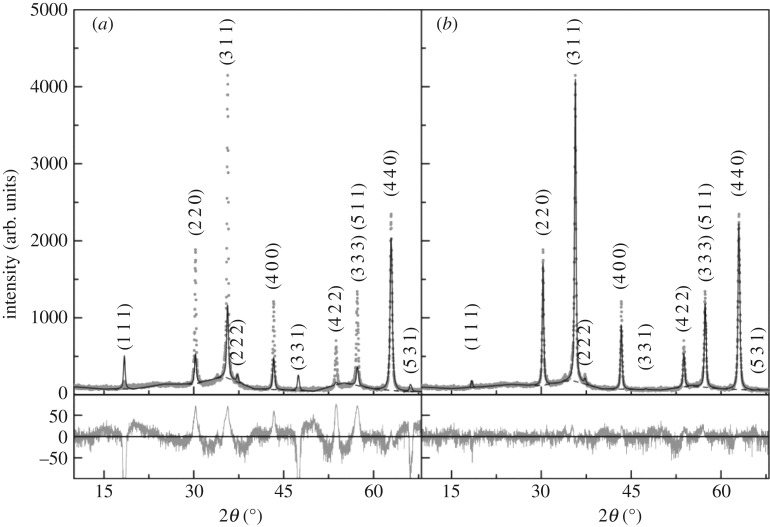
XRD pattern of the MgFe_2_O_4_ nanoparticles calcined at 600°C and Rietveld analysis using preferential orientation correction at the (1 1 0) plane, a fitted background represented by a dashed line and considering either direct (*a*) or 82.5% inverted (*b*) spinel structure.

XRD measurements allowed confirming the synthesis of the magnesium ferrite nanoparticles. A calcination process at 600°C was needed to obtain a crystalline phase. All the characteristic peaks for a pure crystalline phase of magnesium ferrite spinel [19,42] marked by their indices (MgFe_2_O_4_, space group F d -3 m:1, CIF 1011245), are shown in figure 1. For Rietveld analysis using FullProf software [43], the background was defined by a linear interpolation between a set of points at fixed scattering angles, but with fitted intensities. It was impossible to obtain good fits when considering a direct spinel structure of type Mg^Td^Fe_2_^Oh^O_4_ as indicated in CIF 1011245. As the peaks (2 2 0) and (4 4 0) have increased intensities when compared with that of the main peak, (3 1 1), inclusion of preferential orientation correction at (1 1 0) plane [36] resulted in a small but insufficient improvement (electronic supplementary material, table S1). Considering that the spinel structure has a cation ordering according to Mg^Td^_(1−i)_Fe^Td^_i_Mg^Oh^_i_Fe^Oh^_(2−i)_O_4_, the Rietveld optimization resulted in a reasonable fit for a degree of inversion, *i*, of 0.897, with *χ*^2^ = 2.92 and an *R*_F_ factor of 6.8. The use of preferential orientation correction improved the fitting quality parameters to *χ*^2^ = 2.35 and *R*_F_ = 5.03, with *i* = 0.825. The corresponding average size is 33 nm.

The obtained values for the degree of inversion are within the reported values of 0.56 [44] and 0.9 [45], and depend on the preparation method and crystallite size, as it was demonstrated for the case of ZnFe_2_O_4_ [46].

#### 3.1.2. Scanning electron microscopy

**Figure 2.**
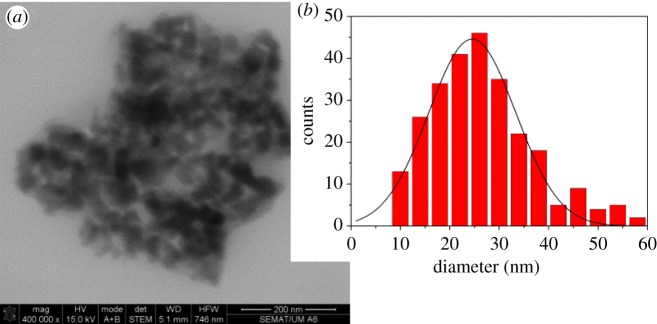
(*a*) STEM image of MgFe_2_O_4_ nanoparticles. (*b*) Particles size histogram of image A and fitting to a Gaussian distribution.

STEM images of the nanoparticles prepared by the procedure here described revealed generally spherical nanoparticles uniform in size, with a size distribution of 24.5 ± 8.7 nm (figure 2). This size compares well with the ones reported by Chandradass *et al.* [14] who prepared nanopowders with 19.6 ± 2 nm diameter (although by a different method, the reverse microemulsion), and also by Kanagesan *et al.* [15] who obtained nanoparticles with diameters around 20 nm. Maensiri * et al.* [42] also prepared MgFe_2_O_4_ nanoparticles (incorporated in electrospun polyvinylpyrrolidone (PVP) nanofibres) with sizes increasing from 15 ± 4 nm to 24 ± 3 nm, when the calcination temperature was raised from 500 to 800°C. Here, the size distribution obtained by STEM is roughly in accordance with the one estimated by XRD.

#### 3.1.3. UV–visible absorption spectra

The UV–visible absorption (or transmission) spectrum of the synthesized magnesium ferrite nanoparticles allows obtaining the optical band gap using a Tauc plot [47], which corresponds to

3.1 $${\left(\alpha h\nu \right)}^{n}\propto (h\nu -E_{g}),$$

where *α* is the absorption coefficient that is proportional to the absorbance, *n* is an exponent that depends on the nature of the transition (being *n* = 2 for a direct semiconductor and *n* = 1/2 for an indirect one), and *E*_g_ is the optical band gap.

**Figure 3.**
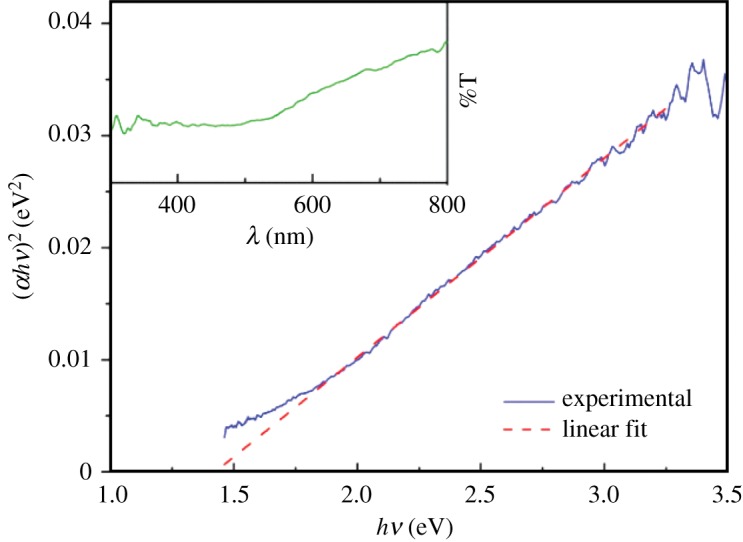
Tauc plot for magnesium ferrite nanoparticles. Inset: UV–visible transmission spectra of NPs dispersed in water.

A linear relation was only obtained for *n* = 2 (figure 3) indicating that MgFe_2_O_4_ behaves as a direct semiconductor.

A band gap of 1.41 eV was estimated from the intercept of Tauc plot (figure 3), in good agreement with the results of Manikandan *et al.* [48] but somewhat lower than the value of 2.0 eV reported by Kim *et al*. [49].

#### 3.1.4. Magnetic properties

**Figure 4.**
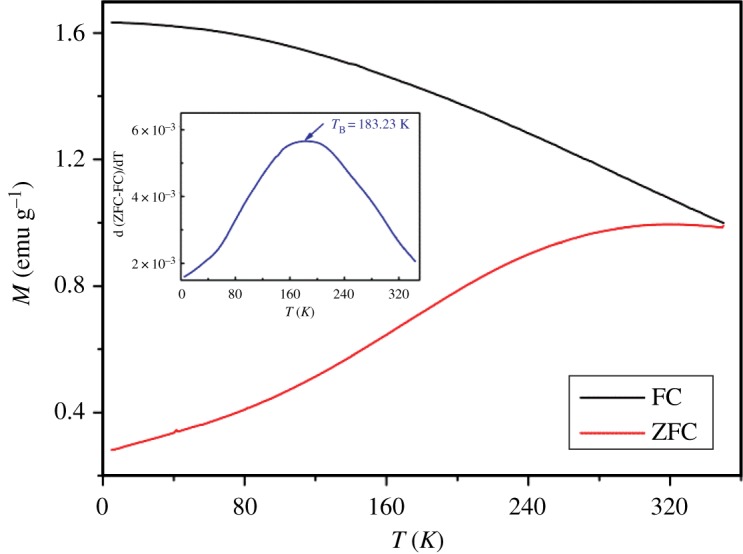
Temperature dependence of the magnetization (ZFC and FC) of MgFe_2_O_4_ nanoparticles over the temperature range 5–350 K, with H = 50 Oe. Inset: Temperature derivative of the difference between ZFC and FC magnetization curves.

Figure 4 displays the temperature dependence of the magnetization measured with zero field cooled (ZFC) and field cooled (FC) curves in the temperature range from 5 to 350 K. The blocking temperature (*T*_B_) distributions of MgFe_2_O_4_ nanoparticles were determined by the temperature derivative of the difference between the ZFC and FC magnetization curves (inset of figure 4). Here, the maximum (*T*_max_ = 183.23 K) corresponds to the blocking temperature of the main size distribution [50]. This method has shown to be more accurate than the one that considers *T*_B_ as the maximum of the ZFC curve [51], as the dipolar interactions result in a shift of the ZFC maximum to higher temperatures.

**Figure 5.**
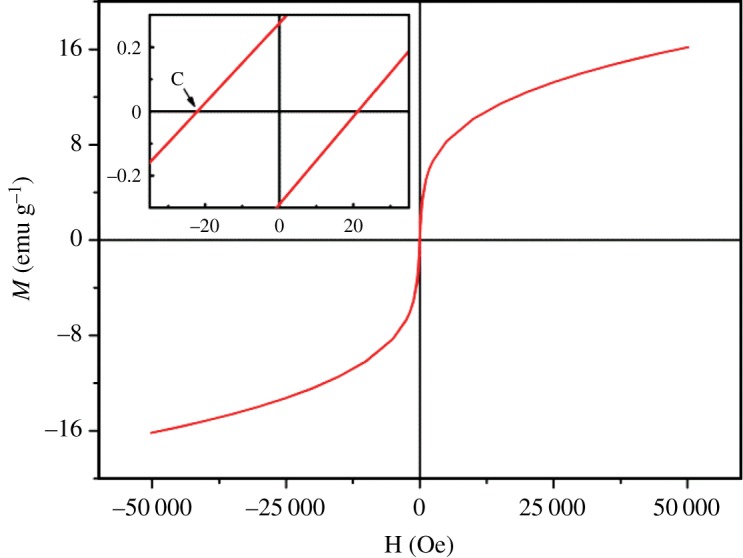
Magnetization hysteresis loop of MgFe_2_O_4_ NPs measured at room temperature. Inset: Enlargement of the loop, in the low field region.

Figure 5 shows the magnetization hysteresis cycle of magnesium ferrite nanoparticles. The maximum magnetization value at 50 kOe, 16.2 emu g^−1^, is slightly smaller than the one obtained by Maensiri *et al.* [42] for MgFe_2_O_4_ nanoparticles incorporated in electrospun PVP nanofibres also calcined at 600°C, but higher than those reported by Kaur & Kaur [16] for NPs prepared by different chemical methods (including coprecipitation). As previously observed by Chandradass *et al.* [14] and Maensiri *et al.* [42], a saturation was not attained in the magnetization hysteresis loop, maybe due to the presence of surface defects. Chandradass *et al*. [14] prepared magnesium ferrite NPs with a maximum magnetization of *M*_s_ = 12.9 emu g^−1^ also by coprecipitation. Maensiri *et al.* [42] reported that calcination at higher temperatures (700–800°C) of MgFe_2_O_4_/PVP nanocomposites causes an increase in the maximum magnetization, but also in both *H*_c_ and *M*_r_, with the loss of superparamagnetism of the NPs (for superparamagnetic nanoparticles, *M*_r_/*M_s_* < 0.1).

A decrease of the magnetization of oxide nanoparticles can be caused by surface effects, like a surface disordered or a magnetically dead surface layer [52]. Moreover, the relative amount of Fe^3+^ ions at A (*T*_d_) and B (*O*_h_) positions in the nanocrystal may strongly decrease the maximum magnetization, because the spins of ions in A and B sites magnetize oppositely the corresponding sublattices in the incomplete inverse spinel structure [53].

Nevertheless, the nanoparticles here obtained stand out considering the low coercive field (*H*_c_) and residual magnetization (*M*_r_), indicating that most of the nanoparticles show a superparamagnetic behaviour (*M*_r_/*M_s_* = 0.02, table 1).

**Table 1.** Coercive field (*H*_C_), maximum magnetization (*M*_s_), remnant magnetization (*M*_r_) and ratio *M*_r_/M_S_ for magnesium ferrite NPs. SQUID data available at http://dx.doi.org/10.5061/dryad.482q2rh [54].

*H*_C_ (Oe)	*M*_S_ (emu g^−1^)	*M*_r_ (emu g^−1^)	*M*_r_/*M*_S_
22.1	16.16	0.26	0.02

The critical size (in zero or a small field), *D*, for superparamagnetic behaviour can be obtained by [55]

3.2 $$D\;=\;\sqrt[{3}]{\frac{6k_{B}T\ln(t_{m}f_{0})}{\pi K}},$$

where *K* is the anisotropy constant, *f*_0_ is the frequency constant (10^9^ Hz), *k*_B_ is the Boltzmann's constant, *T* the absolute temperature and *t*_m_ is the time of measurement. An anisotropy constant of 0.61 × 10^5^ erg cm^−3^ is reported for MgFe_2_O_4_, [56], leading to a critical size for superparamagnetic behaviour of about 33 nm, at room temperature. Thus, the size of MgFe_2_O_4_ nanoparticles here obtained (both by XRD and SEM) is in accordance with the superparamagnetic behaviour predicted by SQUID measurements.

The inductive heating capability of magnetic nanoparticles, under an alternating current magnetic field, is directly proportional to the area of the hysteresis cycle and thus rises as the coercive field is increased, decreasing with the diminution of the maximum magnetization [57]. Here, the significant coercive field (22.1 Oe, table 1) compensates the lower maximum magnetization of MgFe_2_O_4_ nanoparticles when compared with that of other ferrites (nickel, manganese, iron or cobalt) [33,34,50], and points to a possible application of these nanoparticles in magnetic hyperthermia therapies. The negative effect of the lower *M*_s_ value of MgFe_2_O_4_ on the inductive heating capability can also be compensated by the occurrence of small clusters of magnetic nanoparticles that originate an additional and significant heating capacity, enough to reach therapeutic temperatures, as already reported [58].

Comparing with transition metal ferrite nanoparticles previously incorporated in solid and aqueous magnetoliposomes (namely, nickel ferrite and manganese ferrite) [33–35], magnesium ferrite nanoparticles have the advantage of guaranteed biocompatibility [9,10], not compromising the superparamagnetic behaviour. Considering magnetite, despite the reported biocompatibility, it has been shown that these nanoparticles are sensitive to oxidation, being transformed into maghemite in the presence of oxygen [59]. Moreover, it has been described that iron oxide nanoparticles induce the production of reactive oxygen species in mammalian cells [60], causing severe DNA and protein damage and inflammatory responses [61,62]. Therefore, magnesium ferrite nanoparticles can be advantageous, avoiding these undesirable effects.

## 4. Characterization of magnetoliposomes

### 4.1. Dynamic light scattering and scanning electron microscopy measurements

Two types of magnetoliposomes were obtained, solid magnetoliposomes (SMLs) and AMLs. The biocompatibility of magnetoliposomes (containing MnFe_2_O_4_ nanoparticles) of the same lipids and prepared by the same techniques was previously reported [35]. AMLs were prepared by ethanolic injection of the lipids [31] in the aqueous ferrofluid. This methodology was chosen because it is very advantageous for hydrophobic drugs [63] (as is the case of curcumin, exhibiting a very limited solubility in aqueous media), which can be loaded into magnetoliposomes by co-injection. DLS measurements were used to characterize AMLs containing MgFe_2_O_4_ nanoparticles. These systems exhibit diameters of 122 ± 19 nm (size distribution in electronic supplementary material, figure S2), with a low polydispersity (below 0.2). This result is similar to the one reported recently for AMLs of the same lipid containing iron oxide nanoparticles [36], however, being somewhat higher than those of AMLs containing transition metal ferrites [33,34]. For enhanced permeability and retention effect of loaded drugs, the size of magnetoliposomes must be small, and a successful extravasation into tumours has been shown to occur for nanocarriers with sizes below 200 nm [64].

**Figure 6.**
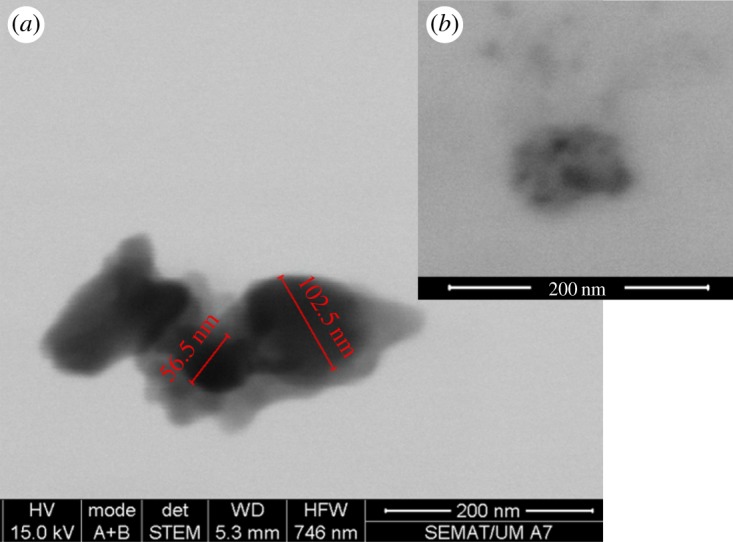
SEM images of SMLs containing magnesium ferrite nanoparticles (obtained with a negative staining).

SMLs were obtained by coverage of a cluster of magnesium ferrite NPs by the phospholipid DPPC, using the method previously developed for Ni ferrite and Mn ferrite nanoparticles [33,34]. Following the same procedure, the formation of the lipid double layer was proven by FRET, Rhodamine B-DOPE labelling the first lipid layer of magnetoliposomes and the second lipid layer containing NBD-C_12_-HPC. Upon double-layer formation, a strong energy transfer is observed from NBD (donor) to Rhodamine B (acceptor), with a FRET efficiency of 73% (electronic supplementary material, figure S3). Using equations (2.3)–(2.5), a donor–acceptor distance of 3.8 nm proves the formation of the lipid bilayer around the nanoparticles cluster, which has a typical thickness between 7 and 9 nm [65]. SEM images of SMLs (obtained with a negative staining) revealed that the synthesized solid magnetoliposomes present diameters slightly above 100 nm (figure 6). Figure 6*a* exhibits several magnetoliposomes containing nanoparticles clusters (with one smaller cluster in the middle). A single magnetoliposome is observed in figure 6*b*. The size obtained is slightly smaller than those of SMLs of DPPC containing MnFe_2_O_4_ and Fe_3_O_4_ nanoparticles [35,36]. As reported previously [35], the interest in using a DPPC bilayer in SMLs is the melting transition temperature of this phospholipid (*T*_m_ = 41°C) [66], approaching the temperatures used in mild hyperthermia therapy. An additional increase in membrane fluidity is expected upon lipid phase transition (from the gel to the liquid crystalline phase), with a potential enhancement in drug release capability of the solid magnetoliposomes.

### 4.2. Incorporation of curcumin in aqueous magnetoliposomes and solid magnetoliposomes

The potential drug curcumin is fluorescent in several polar and non-polar solvents (electronic supplementary material, figure S4). The absorption and emission bands of curcumin can be attributed to the enol-keto tautomer [67], while in water/ethanol and water/acetonitrile mixtures the existence of the diketo tautomer was reported [68,69]. Large spectral red shifts, band enlargement and loss of vibrational structure are observed for the emission in polar media (electronic supplementary material, figure S4), this behaviour being attributed to a strong intramolecular charge transfer mechanism in the excited state, as well as to hydrogen bonding in protic solvents [69].

**Figure 7.**
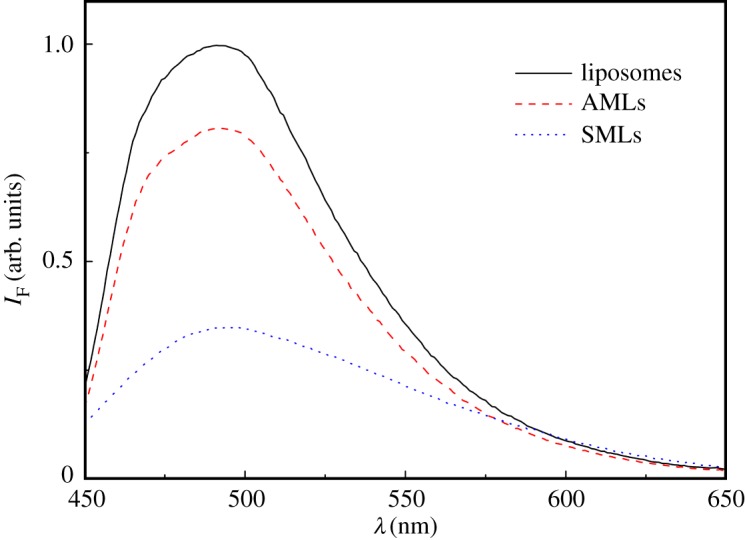
Fluorescence spectra (*λ*_exc_ = 420 nm) of curcumin (3 × 10^−6^ M) in liposomes (without magnetic NPs), AMLs and SMLs containing magnesium ferrite nanoparticles.

Curcumin was loaded into both AMLs and SMLs containing MgFe_2_O_4_ NPs. Figure 7 shows the emission spectra of this dye in AMLs, SMLs and liposomes without magnetic nanoparticles (but with the same concentration of compound), which allow concluding that curcumin is fully incorporated in these nanosystems, as it is non-emissive in aqueous media. It is also possible to observe a quenching effect of the fluorophore emission by the magnetic nanoparticles, much more pronounced in SMLs, similar to what was reported for other potential drugs and different magnetic nanoparticles [34–36]. Also, a small red shift in emission is detected in liposomes and AMLs, when compared with SMLs, indicating a more hydrated environment for curcumin in the latter nanosystems, which was expected considering that SMLs do not contain an interior aqueous volume.

Fluorescence anisotropy measurements (table 2) were performed to confirm that curcumin is fully incorporated in both types of magnetoliposomes, located mainly in the lipid bilayer. First, the anisotropy values are generally high at room temperature, but smaller (as expected) the one measured in the highly viscous solvent glycerol (viscosity around 1000 cP), which is much more viscous than lipid membranes, the latter possessing viscosities of 100–200 cP [71,72]. Also, the transition from the gel phase (at 25°C) to the liquid crystalline phase (at 55°C) of DPPC is detected by curcumin through a decrease in its fluorescence anisotropy; finally, anisotropy values are roughly similar in magnetoliposomes and neat liposomes. However, the anisotropy in SMLs at room temperature is smaller than the one in AMLs or liposomes. Although possible differences in the fluorophore excited-state lifetime can occur, it is known that lipid membrane viscosity decreases from the surface to the interior [73,74]. This can justify the lower value of *r* in SMLs, indicating that curcumin is located deeply in SMLs membranes.

**Table 2.** Steady-state fluorescence anisotropy (*r*) values for curcumin in liposomes (for comparison), AMLs and SMLs. Spectral data available at http://dx.doi.org/10.5061/dryad.482q2rh [54].

	lipid formulation	temperature	*r*^a^
liposomes	Egg-PC	25°C	0.331
	DPPC	25°C	0.287
		55°C	0.119
AMLs	Egg-PC	25°C	0.328
	DPPC	25°C	0.291
		55°C	0.147
SMLs	DPPC	25°C	0.141
		55°C	0.113

^a^Anisotropy of curcumin in glycerol is 0.365 at 25°C [70].

The curcumin encapsulation efficiencies in both AMLs and SMLs are presented in table 3. Values of encapsulation efficiencies are larger than 89%, the lowest *EE*(%) being observed in solid magnetoliposomes. These high encapsulation efficiencies point to a promising use of these nanocarriers for hydrophobic drugs and as agents for simultaneous chemotherapy and magnetic hyperthermia.

**Table 3.** Encapsulation efficiencies (*EE%*) and standard deviations (s.d.) of curcumin in aqueous and solid magnetoliposomes. Original data available at http://dx.doi.org/10.5061/dryad.482q2rh [54].

system	*EE*(%) *±* s.d.
AMLs (Egg-PC)	98.4 ± 1.4
SMLs (DPPC)	89.5 ± 8.0

These results also show the suitability of the ethanolic injection method for the preparation of curcumin-loaded magnetoliposomes. Using the same methodology, Jaafar-Maalej *et al.* [63] reported encapsulation efficiencies in liposomes above 87% for another hydrophobic drug (beclomethasone dipropionate, BDP), attaining 100% in some cases. The *EE*(%) values in table 3 are also similar to the ones previously reported for magnetoliposomes containing new anti-tumour thienopyridine derivatives [35].

Taking advantage of curcumin fluorescence, the non-specific interaction of both types of drug-loaded magnetoliposomes with GUVs was monitored, using GUVs as models of cell membranes [37,38], allowing to investigate the possibility of drug release by fusion. For that purpose, FRET was employed, using curcumin as the energy donor and the hydrophobic dye Nile Red (widely used as lipid probe) [75–78] as the energy acceptor. This is a very favourable donor–acceptor pair, as evaluated by the spectral overlap between curcumin emission and Nile Red absorption (electronic supplementary material, figure S5). Therefore, a high FRET efficiency is expected if the donor–acceptor distance is below 100 Å, this efficiency strongly decreasing with the increase in the donor–acceptor distance [39].

**Figure 8.**
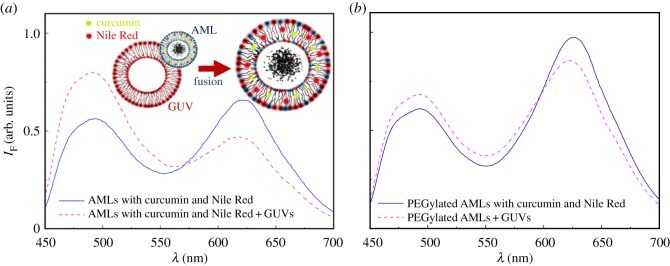
Fluorescence spectra (*λ*_exc_ = 420 nm) of AMLs based on MgFe_2_O_4_ NPs and containing both curcumin and Nile Red, before and after interaction with GUVs. (*a*) AMLs of egg phosphatidylcholine. (*b*) PEGylated AMLs. Inset: Schematic of membrane fusion between AMLs and GUVs.

If magnetoliposomes interact with model membranes (GUVs) and fusion between them occurs, a larger membrane will be created and an increase in the donor–acceptor distance will be verified, with a corresponding decrease in the energy transfer efficiency. This was verified for AMLs, when these nanosystems were loaded with both curcumin and Nile Red (figure 8). Two emission bands, the first due to curcumin fluorescence (*λ*_max_ = 510 nm) and the second due to Nile Red emission (*λ*_max_ = 630 nm) are detected, if only curcumin is excited (figure 8*a*). After interaction with GUVs, an increase in the curcumin (donor) fluorescence band and a decrease of the Nile Red (acceptor) emission band is observed, proving the diminution of FRET efficiency, as a result of membrane fusion.

In order to prevent the adsorption of plasma proteins (opsonization) and promote the shielding from proteolytic enzymes, increasing the retention time in the circulation system [79,80], PEGylation of Egg-PC AMLs was carried out, aiming at obtaining longer-circulating nanosystems in the blood flow. This was achieved by using 5% of DSPE-PEG-2000 in the lipid composition. It has been reported that the coating with PEG is effective in increasing the circulation time of magnetite nanoparticles covered with oleic acid [80] and of magnetoliposomes [6]. However, recently it was also shown that PEGylation can reduce the interaction of magnetoliposomes with cells, with a corresponding decrease in the degree of internalization [81].

Fluorescence anisotropy of curcumin in PEGylated Egg-PC AMLs (*r* = 0.341) is very similar to the one in non-PEGylated magnetoliposomes of the same lipid (table 2). The interaction with GUVs was equally investigated (figure 8*b*). A diminution of FRET efficiency was observed, indicating fusion with GUVs, however, in a lower extent than for non-PEGylated systems. These results point to a higher resistance of PEGylated AMLs to interaction with GUVs, but still maintaining some fusogenic capability.

**Figure 9.**
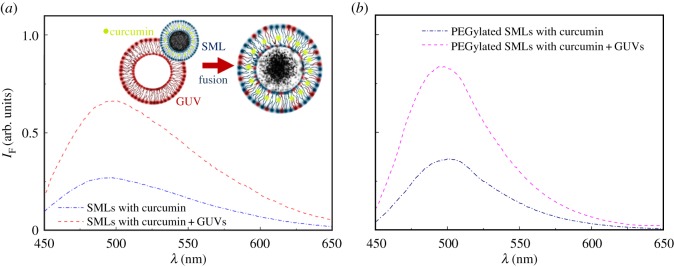
Fluorescence spectra (*λ*_exc_ = 420 nm) of curcumin in SMLs containing MgFe_2_O_4_ nanoparticles, before and after interaction with GUVs. (*a*) SMLs of DPPC. (*b*) PEGylated SMLs. Inset: Schematic of membrane fusion between SMLs (loaded with curcumin) and GUVs.

In the case of solid magnetoliposomes, the emission of curcumin in SMLs incorporating also Nile Red is completely suppressed, as it suffers fluorescence quenching from the nanoparticles and also by energy transfer to the acceptor Nile Red. Therefore, an assay was made with SMLs containing only curcumin. Upon interaction with GUVs, an unquenching effect of the drug fluorescence is detected, proving membrane fusion (figure 9*a*). A similar effect upon interaction with model membranes was detected previously for SMLs containing manganese ferrite nanoparticles [34] and magnetite nanoparticles [36] loaded with other anti-tumour drugs. A small red shift of curcumin fluorescence after interaction with GUVs may indicate, as previously referred, a slightly more hydrated environment felt by the drug after membrane fusion.

Concerning PEGylated SMLs, containing 95% DPPC and 5% DSPE-PEG-2000, the fluorescence anisotropy of curcumin loaded in these systems was determined as *r* = 0.153 at 25°C and *r* = 0.109 at 55°C. Again, the values of *r* are similar to the ones measured in SMLs without PEG (table 2). Upon interaction with GUVs, the unquenching effect of curcumin fluorescence is also detected in PEGylated SMLs (figure 9*b*) and even in a higher degree, which can indicate that the presence of PEG may facilitate the fusion of SMLs with membranes.

Overall, these results show that the aqueous and solid drug-loaded magnetoliposomes, containing superparamagnetic magnesium ferrite nanoparticles, are promising as therapeutic agents, presenting the capability of being guided with a magnetic field to the therapeutic site and releasing the loaded drug by fusion with the cell membrane, while allowing the simultaneous application of magnetic hyperthermia therapy. Moreover, the application of an alternating magnetic field (AMF), causing a local temperature increase by the magnetic nanoparticles, will also promote an increase in fluidity of the lipid membrane of magnetoliposomes, enhancing drug release.

## 5. Conclusion

In this work, magnesium ferrite nanoparticles with sizes around 25 nm and a superparamagnetic behaviour were synthesized. These nanoparticles were used for the preparation of magnetoliposomes, both aqueous (liposomes entrapping magnetic nanoparticles, AMLs) and solid (magnetic nanoparticles covered by a lipid bilayer, SMLs), with sizes below 130 nm.

The model drug curcumin was successfully incorporated in both AMLs and SMLs, with high encapsulation efficiencies. Fluorescence emission measurements (FRET or fluorescence quenching) indicate that curcumin-loaded AMLs and SMLs interact with models of cell membranes (GUVs) by fusion, even when PEGylation is used.

To our knowledge, it is the first time that solid and aqueous magnetoliposomes containing superparamagnetic magnesium ferrite nanoparticles were prepared and their potentialities as drug nanocarriers evaluated. These results point to future applications of magnetoliposomes containing MgFe_2_O_4_ nanoparticles in dual cancer therapy (combining magnetic hyperthermia and chemotherapy).

## Supplementary Material

Electronic Supplementary Material

## Data accessibility

Experimental data are deposited at Dryad Digital Repository: http://dx.doi.org/10.5061/dryad.482q2rh [54].
