# Supplementary material for: Magnetoliposomes containing magnesium ferrite nanoparticles as nanocarriers for the model drug curcumin
Source: R Soc Open Sci. 2018 Oct 17;5(10):181017. doi: 10.1098/rsos.181017 (PMC6227978; doi:10.1098/rsos.181017)
Supplement: Electronic Supplementary Material [file rsos181017supp1.pdf]

Magnetoliposomes containing magnesium ferrite nanoparticles as nanocarriers for the model drug curcumin

Beatriz D. Cardoso,[1] Irina S. R. Rio,[1] Ana Rita O. Rodrigues,[1] Francisca C. T. Fernandes,[1] B. G. Almeida,[1] A. Pires,[2] A. M. Pereira,[2] J. P. Araújo,[2] Elisabete M. S. Castanheira[1] and Paulo J. G. Coutinho[1],*

[1] *Centre of Physics (CFUM), University of Minho, Campus de Gualtar, 4710-057 Braga, Portugal*

[2] *IFIMUP/IN - Instituto de Nanociência e Nanotecnologia, R. Campo Alegre, 4169-007 Porto, Portugal*

Supplementary Information

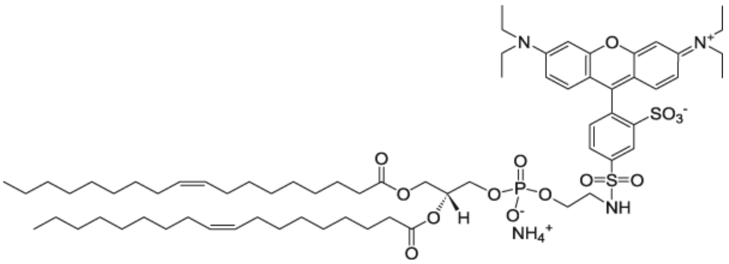

Rhodamine B-DOPE

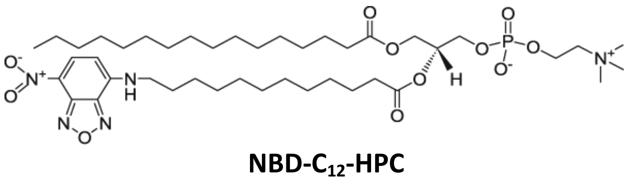

NBD-C_{12}-HPC

Figure S1. Structures of the fluorescent-labelled lipids used in FRET assays.

Results of the Rietveld analysis

Table S1. Selected Rietveld analysis parameters (Overall temperature factor, B_{over}=0)

Analysis	$O_{x,y,z}$ (*)	Preferred orientation (**)	Degree of inversion (*i*)	Size (nm)	R_f	χ^2
**A**	0.3593	No (*r* = 1)	0	20	27.0	30.2
**B**	0.3673	Yes (*r* = 0.46)	0	21	26.8	29.8
**C**	0.3781	No (*r* = 1)	0.897	33	6.8	2.92
**D**	0.3762	Yes (*r* = 0.64)	0.825	33	5.03	2.35

(*) Value in CIF file nr. 11011245 is 0.375

(**) According to March function,[1] for (1 1 0) plane:

$$\left(r^2 \cos^2 \alpha + \frac{\sin^2 \alpha}{r}\right)^{-3/2}$$

where, for a platy habit, α is the acute angle between the scattering vector and the normal to the crystallites.

Size distribution of aqueous magnetoliposomes containing $MgFe_2O_4$ nanoparticles obtained by Dynamic Light Scattering (DLS)

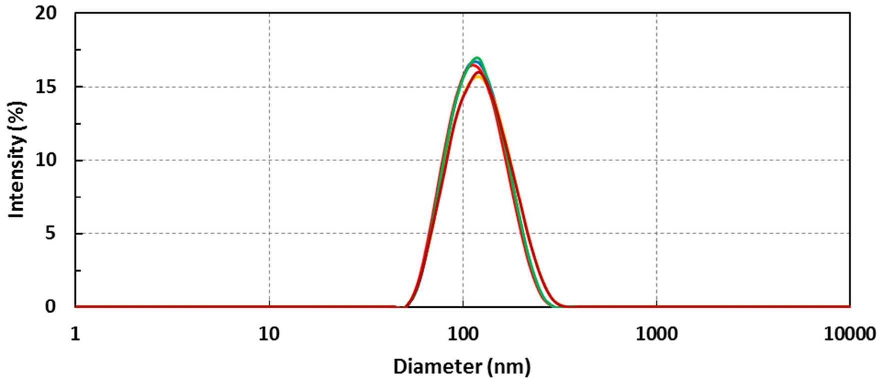

Figure S2. Size distribution (by intensity) obtained by DLS for aqueous magnetoliposomes of egg phosphatidylcholine containing $MgFe_2O4$ nanoparticles (at 25° C).

FRET assay for confirmation of the lipid bilayer in SMLs

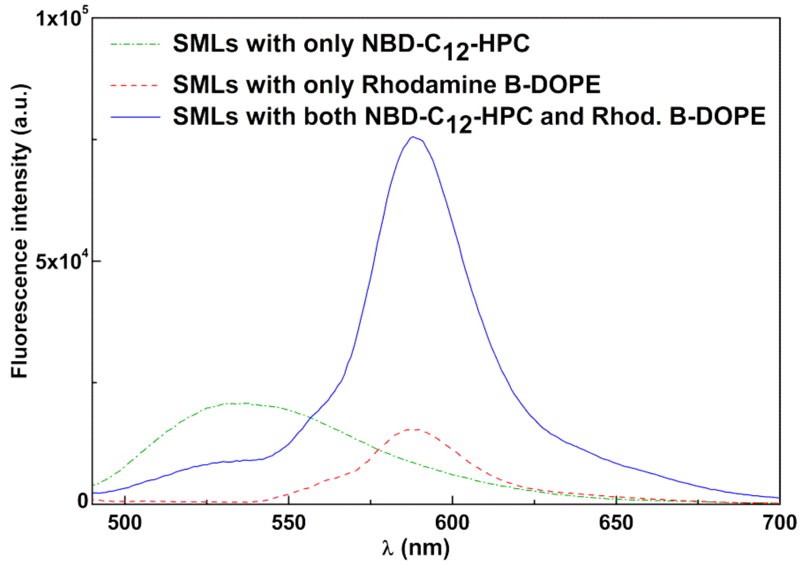

Figure S3. Fluorescence spectra of solid magnetoliposomes (λ_{exc}=470 nm) containing $MgFe_2O_4$ NPs, labeled only with NBD-C_{12}-HPC, labeled only with Rhodamine B-DOPE, and with NBD-C_{12}-HPC (in the outer lipid layer) and Rhodamine B-DOPE (in the inner lipid layer).

Curcumin absorption and fluorescence emission spectra in several solvents

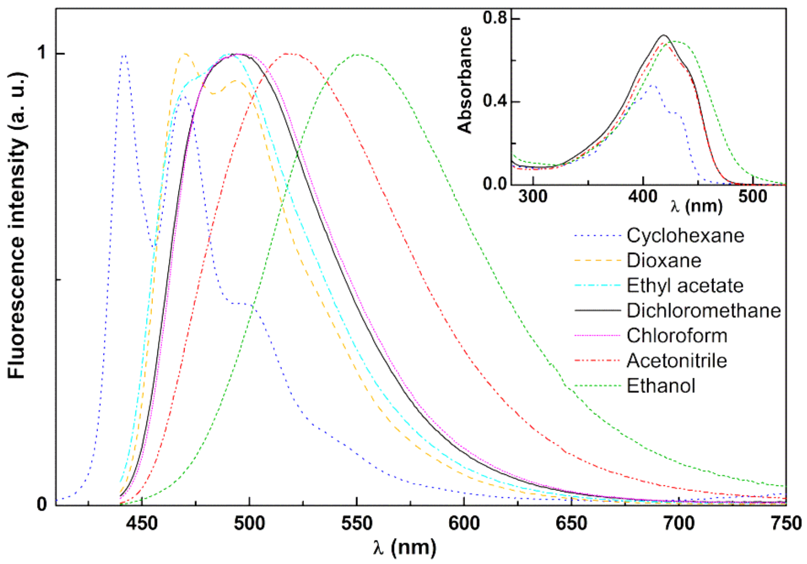

Figure S4. Normalized fluorescence spectra (at peak of maximum emission) of 3×10^{-6} M solutions of curcumin in several solvents (λ_{exc}=410 nm). Insets: Absorption spectra of 1×10^{-5} M solutions (cell path = 1.0 cm).

FRET assays for interaction between magnetoliposomes and GUVs, using curcumin and Nile Red

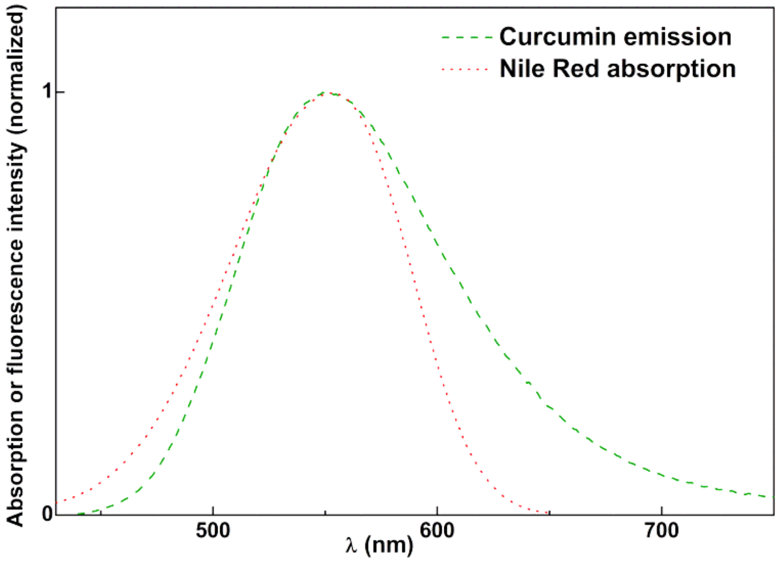

Figure S5. Spectral overlap between curcumin fluorescence emission and Nile Red absorption.

References

1. W. A. Dollase, Correction of intensities for preferred orientation in powder diffractometry: Application of the March model, *J. Appl. Cryst.*, 1986, **19**, 267-272.
